# Supplementary material for: Quantifying Strain and Its Effect on Charge Transport in Ge/Si Core/Shell Nanowires
Source: Adv Sci (Weinh). 2026 Mar 28;13(32):e74995. doi: 10.1002/advs.74995 (PMC13252618; doi:10.1002/advs.74995)
Supplement: Supplementary file 1 — Supporting File: advs74995‐sup‐0001‐SuppMat.docx. [file ADVS-13-e74995-s001.docx]

Supporting Information

Quantifying Strain and its Effect on Charge Transport in Ge/Si Core/Shell Nanowires

*Aswathi K. Sivan, Nicolas Forrer, Aakash Shandilya, Yang Liu, Alexander Vogel, Arianna Nigro, Janica Böhler, Pierre Chevalier Kwon, Artemii Efimov, Ilya Golokolenov, Gerard Gadea, Riccardo Rurali, Andreas Baumgartner, Dominik Zumbühl, and Ilaria Zardo^*^*

**Section S1: Structural characterization of the CS NWs**

In this section we present structural characterization for two additional CS NWs, more specifically for shell thicknesses of 5.5 ± 1.0 and 15.1 ± 3.0 nm. In Figure S1 (a) HR-TEM is presented, indicating the high crystal quality of the samples, whereas for the 15 nm thick shell (b) a poly-crystalline growth is observed, resulting from the relaxation of the strain due to the lattice mismatch of the two materials. EDX line scans (c) highlight the presence of the target materials Si and Ge distributed in the shell and the core, respectively. Please note that panel (b) shows a top-down view instead of a cross-sectional one.


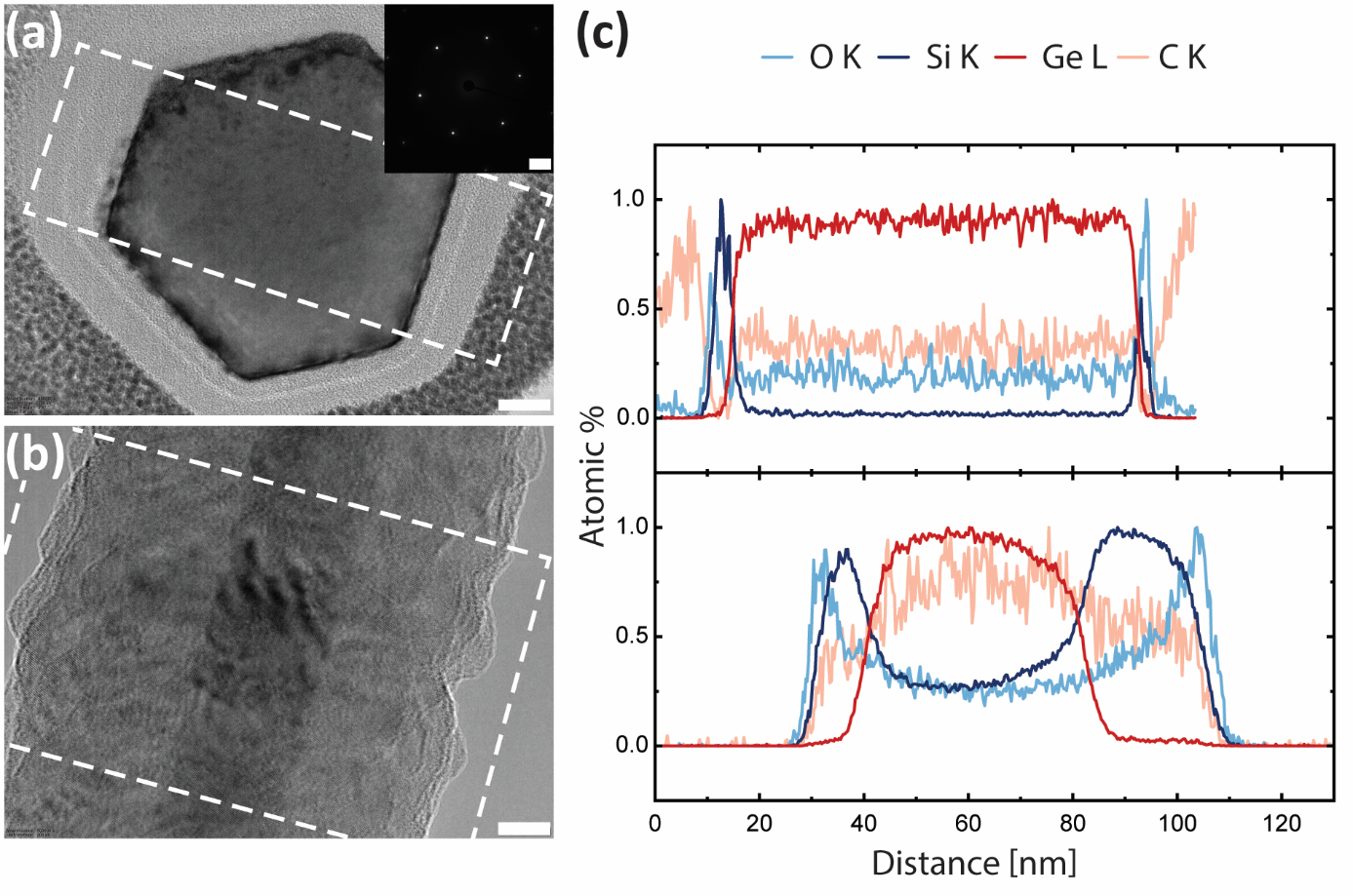


Figure S 1. (a) Cross-sectional and (b) top-down HR-TEM images of Ge/Si CS NWs grown from a 15 nm Au colloid with shell thickness of 5.5 ± 1.0 and 15.1 ± 3.0 nm, respectively. The inset in (a) shows a SAED pattern obtained on the cross-section. The scale bar for the HR-TEM images and for the SAED patterns is 10 nm and 2 nm^-1^ in each panel, respectively. (c) EDX line profiles of the O-K, Si-K, Ge-L, and C-K edges obtained on the HR-TEM images, as indicated in panels (a) – (b).

**Statistics of the core diameters grown with different Au-nanoparticles**

To identify the spread in the core-diameters, we performed STEM analysis on the CS NWs used in the Raman measurements presented in Figure 4 in the main text. The results of this analysis are presented in Figure S2. As expected, we see a spread in the Ge core diameters but we observe that as the Au colloid size increases, the diameter of the Ge core also increases.


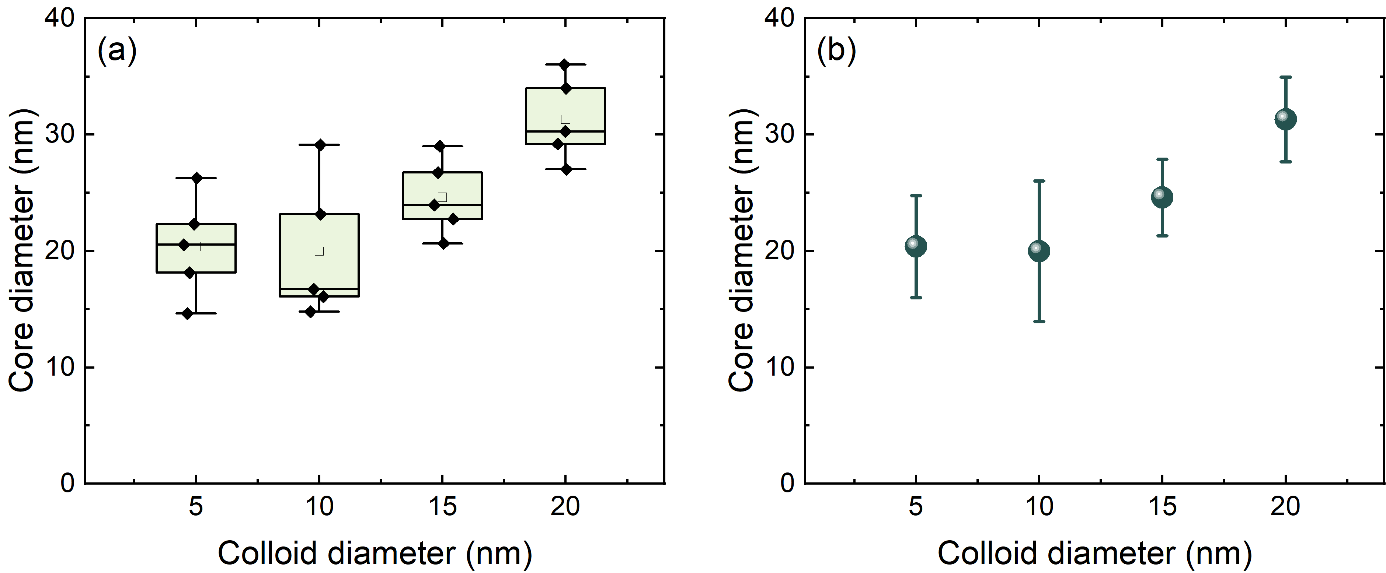


Figure S 2. (a) The spread in the diameters of Ge core as a function of Au colloid size; (b) The average value of Ge core diameter as a function of Au colloid size, the error bars correspond to the standard deviation of the distribution.

**Homogeneity of the shell thickness along the nanowire length**

In order to assess the homogeneity of the Si shell thickness along the nanowires’ length, we have performed STEM measurements of four different exemplary nanowires with a nominal shell thickness of 5.5 ± 1.0 nm (Figure S 3) and extracted the shell thickness along the NW length.

All investigate nanowires are about 1 µm long and exhibit a variation of the shell thickness below 0.8 nm.

**
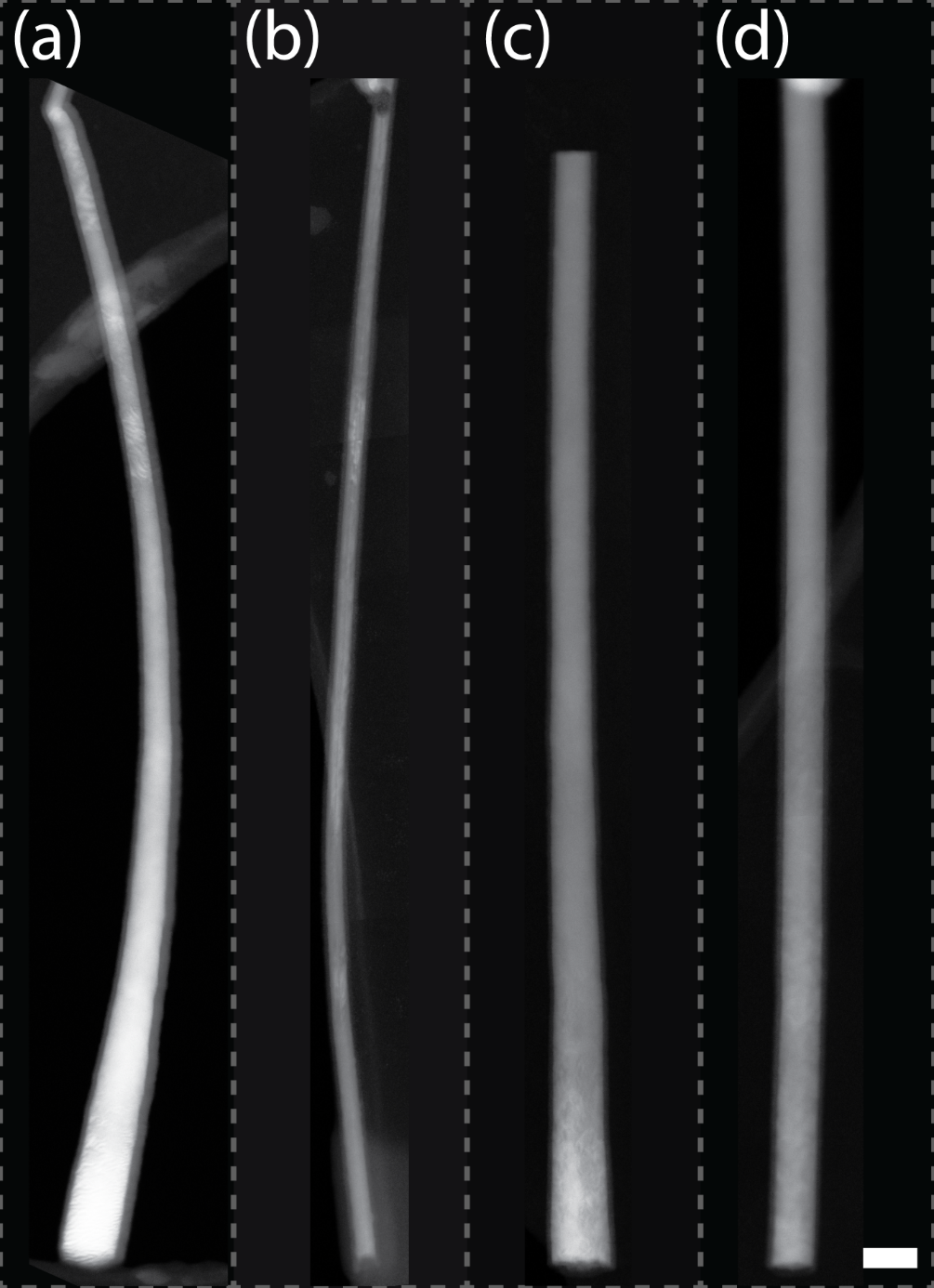
**

Figure S 3. STEM HAADF images of four representative nanowires, where the Ge core and Si shell present a different contrast. The scale bar is 50 nm in each panel.

**Section S2. µ-Raman Spectroscopy:**

**Statistics of the Measurements:**

Figure S4 shows the statistics of the CS NWs with different Si shell thicknesses. To quantify wire-to-wire variability, we measured the Ge–Ge Raman peak for Ge/Si core–shell NWs with Si-shell thicknesses of 5.5 ± 1.0, 6.5± 2.0, and 11 ± 2 nm. For each set of Si shell thickness, the NWs were transferred from the as-grown sample onto a TEM grid prior to Raman on individual NW measurement. These grids were then used for microscopy analysis. For each condition, individual peak positions cluster around the batch mean (dotted line), with the grey band indicating ± SD.





S 4. Wire-to-wire statistics of the Ge–Ge Raman peak for Ge/Si CS NWs with different Si-shell growth times. (a) 5.5 nm Si shell, (b) 6.5 nm Si shell, and (c) 11 nm Si shell; Ge cores grown using 15-nm Au colloids. Red circles are peak positions from individual NWs (error bars from the line-shape fit); the black dotted line marks the mean peak position from each sample, and the grey band shows ± standard deviation. All wires in a panel were taken from the same as-grown sample and measured under identical conditions.

**Raman measurements on a 15.1 nm thick Si shell growth on 15 nm Ge core:**

Extending the Si-shell growth to 15 mins created a 15.1 ± 3.0 nm thick Si shell and produces a red shift of the Ge–Ge Raman peak, consistent with defect-mediated relaxation of the compressive core strain.





S 5 The Raman measurement on a 15.1 ± 3.0 nm Si shell CS NW, here the Raman peak is red shifted maybe attributed to the onset of defect-driven relaxation in 15.1 ± 3.0 nm Si shells.

**Choice of the fit – Fano vs Lorentz function**

In this work, we have used a Lorentz function to fit the Ge-Ge phonon mode for the bare Ge-NWs and a Fano function for the Ge-Ge phonon mode for the Ge/Si CS NWs. The peak is weakly asymmetric, with a longer low-frequency tail. The Fano lineshape accounts for the interference between the discrete phonon and the hole continuum.





Figure S 6. Fano (black) reproduces the slight asymmetry and low-frequency tail of the Ge peak; the Lorentzian (blue) underestimates intensity on the low-frequency side.

**Section S3. Mobility measurements:**

**Simulation of capacitance between NWs and back gate**

As mentioned earlier, both ends of each NW are connected by Ti/Pd ohmic contacts. Since the NW itself is conductive, it, together with its contacts, effectively forms a dumbbell-shaped metallic structure above the gate. The P-doped Si substrate is also conductive and serves as a global back gate. To evaluate the gate–NW coupling, we perform COMSOL simulations of the capacitance between this metallic structure and the back gate, modelling the NW and its contacts as a single electrode for computational simplicity. The geometric parameters—including the NW length between contacts (L), contact width (W), and NW diameter (D)—are extracted from SEM images. Based on TEM and EDX analyses, the Ge core radius is taken as two-thirds of the total NW radius, with the remaining one-third corresponding to the Si shell. The ohmic contact length is set to 4 μm, sufficiently long compared with the active NW segment, and the SiO₂ thickness on the substrate is fixed at 300 nm. For each device, these geometric parameters are determined individually, and the model is then used to simulate the corresponding capacitance prior to extracting the mobility. Figure S7 shows a schematic top view and cross-section of the mobility device used in the simulation: the dark blue region corresponds to the P-doped conductive substrate, the light blue layer represents the 300 nm SiO₂ on the substrate, and the grey boxes indicate the Ti/Pd ohmic contacts. In the NW, the orange region denotes the Ge core, while the green region corresponds to the Si shell.


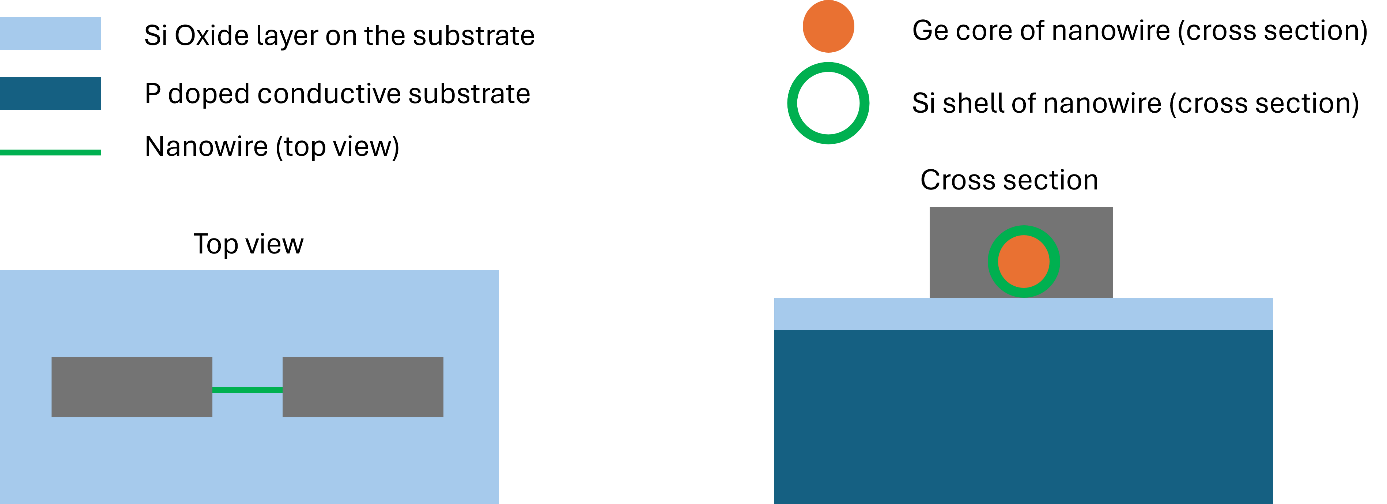


Figure S 7. Schematic top view and cross-section of the mobility device used in the simulation. The light grey region represents the P-doped conductive substrate, while the dark grey layer corresponds to the 300 nm SiO₂ on the substrate. The green boxes indicate the Ti/Pd ohmic contacts. For the NW, the crimson region denotes the Ge core, and the blue region denotes the Si shell

**Details of NW Parameters**

| Sr. No. | Length (nm) | Diameter (nm) | Mobility (cm2/Vs) | Pinch-off voltage (V) | Contact resistance (kΩ) | Total resistance ($V_{G}=0$) (kΩ) | Mean free path  1V above depletion (nm) |
| --- | --- | --- | --- | --- | --- | --- | --- |
| 1 | 1,280 | 40 | 7,000 ± 2,000 | 4.6 ± 0.9 | 18.2 ± 1.5 | 38 | 126 ± 36 |
| 2 | 505 | 53 | 2,000 ± 200 | 6.2 ± 0.3 | 16.2 ± 1.3 | 50 | 23 ± 2 |
| 3 | 890 | 35 | 1,500 ± 100 | 8.2 ± 0.5 | 21.1 ± 0.4 | 33 | 23 ± 1 |
| 4 | 510 | 43 | 7,900 ± 1,600 | 7.1 ± 0.2 | 7.2 ± 0.3 | 11 | 100 ± 20 |
| 5 | 375 | 43 | 1,100 ± 100 | 8.2 ± 0.6 | 19.7 ± 1.4 | 50 | 12 ± 1 |
| 6 | 1,030 | 32 | 7,800 ± 1,600 | 7.5 ± 0.2 | 12 ± 1 | 18 | 128 ± 26 |
| 7 | 590 | 53 | 1,100 ± 100 | 7.1 ± 0.4 | 16.9 ± 1.2 | 50 | 17 ± 1 |
| 8 | 1,570 | 47 | 1,300 ± 200 | 0.2 ± 0.3 | 57 ± 2 | 5,000 | 26 ± 4 |
| 9 | 640 | 30 | 9,400 ± 1,800 | 11.9 ± 0.5 | 7.2 ± 0.7 | 8 | 127 ± 24 |
| 10 | 370 | 23 | 8,400 ± 1,500 | 14.2 ± 0.2 | 5.4 ± 0.6 | 6 | 80 ± 14 |
| 11 | 550 | 27 | 17,500 ± 5,200 | 11.1 ± 0.2 | 8.3 ± 0.8 | 9 | 210 ± 62 |
| 12 | 480 | 22 | 9,000 ± 3,000 | 13.4 ± 0.1 | 10 ± 0.8 | 12 | 100 ± 33 |
| 13 | 500 | 25 | 7,800 ± 1,800 | 12.6 ± 0.3 | 7.2 ± 0.6 | 9 | 90 ± 20 |
| 14 | 660 | 22 | 6,500 ± 1,600 | 7.5 ± 0.5 | 26 ± 1 | 32 | 82 ± 20 |
| 15 | 420 | 22 | 13,600 ± 3,200 | 9 ± 0.1 | 17 ± 2 | 18 | 133 ± 31 |
| 16 | 530 | 20 | 25,400 ± 4,500 | 9 ± 0.1 | 14.5 ± 1.5 | 18 | 275 ± 49 |
| 17 | 300 | 35 | 9,100 ± 2,100 | 4.8 ± 0.2 | 6.7 ± 0.6 | 12 | 80 ± 18 |
| 18 | 450 | 26 | 2,000 ± 1,100 | 1.2 ± 0.8 | 25 ± 3 | 184 | 21 ± 11 |
| 19 | 500 | 26 | 10,800 ± 2,300 | 1.8 ± 0.2 | 10 ± 1 | 39 | 120 ± 25 |
| 20 | 400 | 20 | 9,100 ± 2,700 | 5.1 ± 0.3 | 6.5 ± 0.2 | 13 | 79 ± 23 |
| 21 | 550 | 28 | 6,400 ± 1,600 | 3.3 ± 0.2 | 14 ± 1 | 26 | 69 ± 17 |
| 22 | 550 | 29 | 10,800 ± 2,300 | 5.2 ± 0.2 | 5.7 ± 0.5 | 12 | 118 ± 25 |

Table S 1. Detailed parameters of the measured NWs

Table summarizes the parameters of all 22 NWs measured in this work. The NW length is defined as the distance between the two ohmic contacts, which was extracted from SEM images together with the diameter. The total resistance is measured at $V_{G}=0$. The NW–back gate capacitance was simulated using COMSOL, which was used to extract the pinch-off voltage, contact resistance, and carrier mobility by fitting to Eq. 1. The mean free path is estimated as detailed below at a gate voltage of 1 V before depletion.

**Mean free path**

Using a simple Drude-Sommerfeld model, the mean free path for one-dimensional (1D) transport is given

$l=\mu_{h}n\frac{\pi\hbar}{2e}$ ,

while for three-dimensional (3D) transport is

$l=\mu_{h}\sqrt[3]{n}\frac{\hbar\sqrt[3]{3\pi^{2}}}{e}$ .

Here, $n$ is the charge carrier density, which depends on the gate voltage according to

$n=\frac{c(V_{G}-V_{th})}{e}$ ,

where $c$ is the channel capacitance per unit length for the 1D case and per unit volume for the 3D case. Using the parameters of our NWs, we find that the mean free paths obtained from the 1D and 3D models are quite similar and in good agreement. The mean free path calculated using the 1D expression is listed in Table S1 and plotted as a function of mobility in Figure S8 at a gate voltage of 1 V before pinch-off using a simple Drude model. Notably, farther away from pinch-off, the extracted mean free path can exceed the channel length. In principle, this could imply the emergence of quantized conductance which, however, is not observed in the measurements.


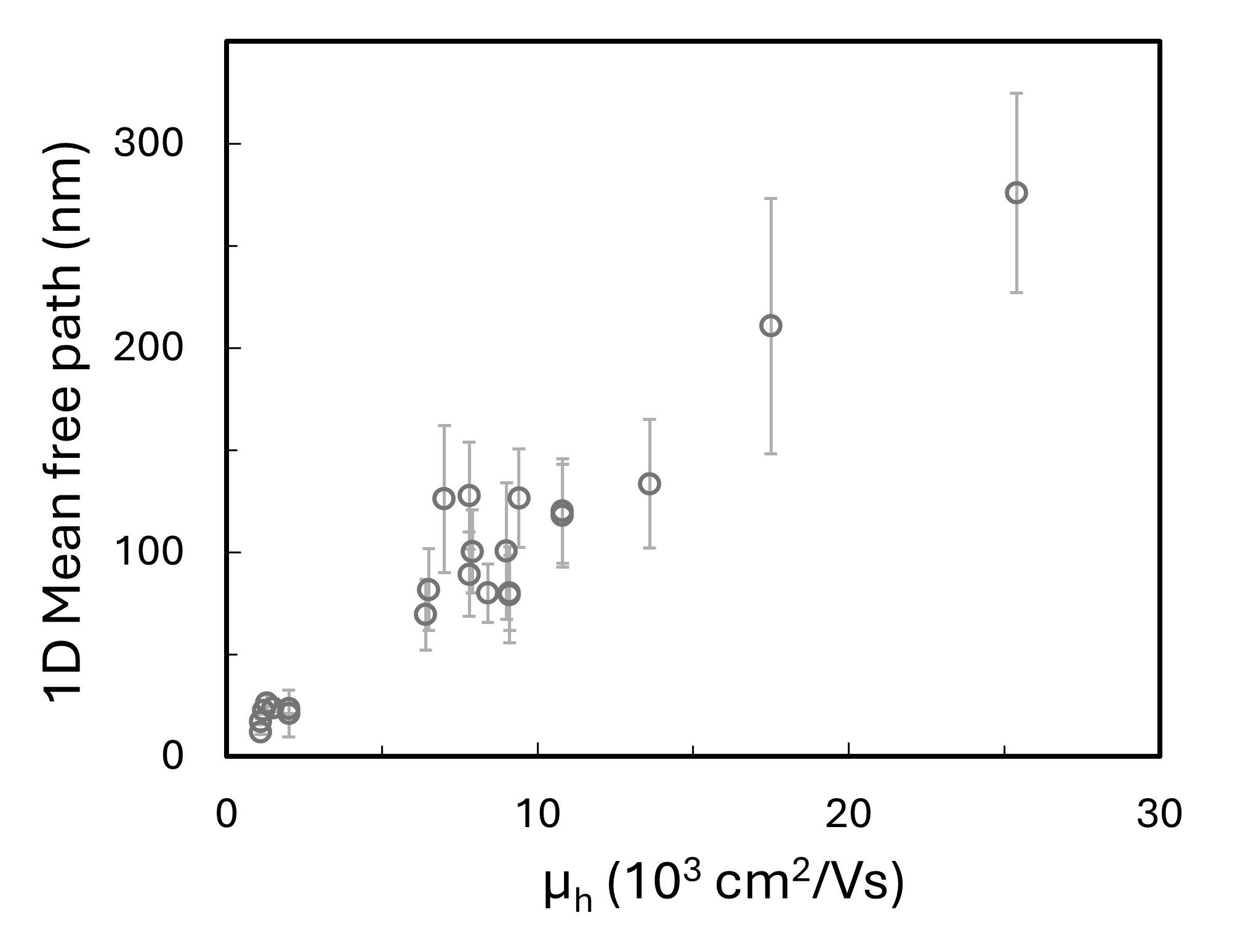


Figure S 8. 1D mean free path of the 22 NWs as a function of carrier mobility. The mean free path values are obtained using the parameters of our NWs and are listed in Table S1. The results show good agreement with those derived from the three-dimensional (3D) model.
